# Impact of etravirine on hospitalization rate between 2005 and 2011 among heavily treated HIV-1-infected individuals on failing regimens

**DOI:** 10.1186/s12879-018-3231-5

**Published:** 2018-07-11

**Authors:** Valérie Potard, Cécile Goujard, Marc Antoine Valantin, Jean Marc Lacombe, Rima Lahoulou, Arnaud Chéret, Pierre Marie Girard, Dominique Costagliola, French Hospital Database on HIV (FHDH - ANRS CO4)

**Affiliations:** 1 Institut Pierre Louis d’épidémiologie et de Santé Publique (IPLESP UMRS 1136), Sorbonne Universités, INSERM, UPMC Univ Paris 06, F75013 Paris, France; 2 INSERM-TRANSFERT, Paris, France; 3 AP-HP, Service de Médecine Interne et d’Immunologie clinique, INSERM CESP, Hôpital de Bicêtre, Univ Paris Sud, Le Kremlin-Bicêtre, France; 4 AP-HP, Service des Maladies Infectieuses et Tropicales, Hôpital Pitié-Salpêtrière, Paris, France; 5 JANSSEN, Issy-les-Moulineaux, France; 6 AP-HP, Service des Maladies Infectieuses et Tropicales, Hôpital Saint-Antoine, Paris, France; 7 MSD France, Courbevoie, France; 8 Inserm UMR_S 1136, 56 Bd Vincent Auriol, 75646 Paris Cedex 13, CS 81393 France

**Keywords:** Antiretroviral experienced HIV-infected individuals, Etravirine, Hospitalization rates

## Abstract

**Background:**

Etravirine (ETR), a non-nucleoside reverse transcriptase inhibitor (NNRTI) available in France since 2006, is indicated for antiretroviral-experienced HIV-infected adults, in combination with a ritonavir-boosted protease inhibitor (PI). To assess its clinical impact in routine care, we compared hospitalization rates according to ETR + PI prescription or not, among heavily treated HIV-1 infected individuals on failing regimens between 2005 and 2011.

**Methods:**

From the French Hospital Database on HIV (ANRS CO4), we selected heavily treated individuals (prior exposure to at least 2 nucleoside reverse transcriptase inhibitor (NRTI), 2PI and 1 NNRTI) with viral load (VL) > 50 copies/mL who started a new antiretroviral (ARV) regimen between 2005 and 2011. Using an intention-to-continue-treatment approach, hospitalization rates were calculated for the individuals who received ETR + PI, during the months after initiating ETR + PI (ETR + PI) or for the individuals who received ETR + PI, in the months before ETR + PI initiation and for the individuals who never received ETR + PI (no ETR + PI). hospitalization from an AIDS-defining cause and hospitalization from a non-AIDS defining cause rates were also calculated. Poisson regression models were used to compare the incidences between the two groups, with adjustment for potential confounders.

**Results:**

Of 3884 patients who met the inclusion criteria, 838 (21.6%) received ETR + PI. During 13,986 person-years (P-Y) of follow-up, there were 2484 hospitalizations in 956 individuals. The hospitalization rates per 1000 P-Y were 169.0 among individuals exposed to ETR + PI and 179.3 among those not exposed to ETR + PI. After adjustment, the respective hospitalization rates were 148.8 and 186.7 per 1000 P-Y, with an estimated relative risk of 0.80 (95%CI: 0.71–0.90), AIDS hospitalization rates were 11.5 and 22.7 per 1000 P-Y, with an estimated relative risk of 0.51(95%CI: 0.39–0.66) and non-AIDS hospitalization rates were 139.5 and 152.2 per 1000 P-Y, with an estimated relative risk of 0.92 (95%CI: 0.80–1.05).

**Conclusions:**

Between 2005 and 2011, access to ETR + PI was associated with a 20% reduction in the hospitalization rate among heavily treated HIV-1-infected individuals. This reduction was mainly due to a reduction in the AIDS hospitalization rate.

## Background

The primary objective of antiretroviral therapy (ART) is to achieve and maintain HIV viral load (VL) below the detection limit of current assays, in order to promote immune reconstitution and to avoid the accumulation of resistance mutations [1, 2]. The rate of VL suppression on combination antiretroviral therapy (cART) has gradually increased over the past 15 years [3]. However, rates of virologic failure to the 3 original classes, nucleoside reverse transcriptase inhibitors (NRTI), non-nucleoside reverse transcriptase inhibitors (NNRTI) and protease inhibitors (PI) remained substantial, and new drugs were therefore needed [4, 5]. Efficacy of new drugs, including drugs in new classes, has been reported in clinical trials including individuals with triple class virologic failure [6], but the impact of these new drugs on clinical outcome has not been examined. The aim of this study was to assess the impact of introduction of etravirine (ETR), a NNRTI available since 2006 for use in combination with a boosted PI, on the incidence of severe morbidities, as reflected by the hospitalization rate, among heavily treated HIV-1-infected individuals on failing regimens.

## Methods

### Data source

The French Hospital Database on HIV (FHDH) is a hospital-based open multicentre cohort in which inclusions have been ongoing since 1989 [7]. It includes data from 70 French general or university hospitals distributed throughout France. Individuals are eligible if they have documented HIV-1 or HIV-2 infection and give their written informed consent to participate. Data are collected prospectively by trained research assistants on standardized forms which include demographic characteristics, biological markers such as the CD4 cell count and plasma HIV RNA level, the date and type of AIDS and non AIDS-defining events, antiretroviral treatments, and the date and causes of death.

### Study population

From the FHDH ANRS CO4 cohort, we selected heavily treated HIV-1-infected individuals (prior exposure to at least 2 NRTI, 2 PI and 1 NNRTI), with VL > 50 copies/mL who were starting a new drug (either a PI, or a NNRTI or an integrase inhibitor, or an entry inhibitor or a fusion inhibitor) between 2005 and 2011. The date of inclusion or baseline was the date the first such drug was prescribed during the study period. The study period was chosen in order to assess the risk of hospitalization before and after ETR became available in France. Individuals with less than 6 months of follow-up after the prescription of a first new drug and no available CD4 cell count within 6 months before the date of inclusion were excluded.

### Statistical analysis

We compared follow-up with or without access to ETR + PI and a given participant can contribute to only the ETR + PI or to only the no ETR + PI or both, over the course of the study. As a given individual could initially be unexposed and then exposed to ETR + PI during the study period, methods taking into account the time-dependent nature of the variable of interest were used for descriptive analyses comparing characteristics of exposed and unexposed individuals and to analyze the impact of ETR + PI exposure on the hospitalization rate. For each individual, the follow-up period was divided into months. To compare the characteristics of exposed and unexposed individuals, a weighted Chi-square test was used, the weight being the duration of follow-up within each month.

The primary endpoint was the rate of hospitalization. Secondary endpoints were the two individual components of the primary endpoint, namely hospitalization from an AIDS-defining cause and hospitalization from a non-AIDS defining cause. We considered only hospitalizations lasting > 24 h, that were not due to pregnancy or to regular medical follow-up or medical examinations. Using an intention-to-continue-treatment approach, the number of hospitalizations and person-times were calculated for each calendar month for the individuals who received ETR + PI, during the months after initiating ETR + PI (ETR + PI) or for the individuals who received ETR + PI, in the months before ETR + PI initiation and for the individuals who never received ETR + PI (no ETR + PI). Individuals who received ETR + PI could also received another ARV at the same time. Adjusted incidence rates and relative risks (RR) were obtained from Poisson regression models in order to compare hospitalization rates between the exposed and unexposed groups, taking into account the following potential confounders: gender and transmission group (men who have sex with men, injecting drug users, other men, other women), geographic origin (sub-Saharan Africa, others), age, HCV co-infection status, the nadir CD4 cell count (< 50, 50–100, 100–200, ≥ 200 /mm^3^), the baseline CD4 cell count (< 200, 200–350, 350–500, ≥ 500 /mm^3^), baseline viral load (50–500, 500–5000, 5000–30,000, 30,000–100,000, ≥ 100,000 copies/mL), AIDS status at baseline, pneumocystis jiroveci prophylaxis (dapsone, cotrimoxazole or pentamidine aerosol) at baseline and number of previous antiretroviral drugs (ARV). Boosted ritonavir was not counted as an additional drug. In addition, as the centre size can influence quality of care, the analyses were adjusted for total follow-up (person-years (P-Y)) of heavily treated patients in each centre, using a four-level variable according to quartiles distribution (< 140 P-Y, 140–240 P-Y, 240–400 P-Y, ≥ 400 P-Y). A second model was further adjusted on exposure to NRTI and to raltegravir (RAL), a new drug in the new integrase inhibitor class, also made available in 2006 for individuals on failing regimens, as a time-dependent variable, to disentangle the respective roles of access to ETR + PI and to RAL. As improvements in individual management during the study period, rather than exposure to ETR + PI, could also explain observed changes, we conducted a sensitivity analysis including the period in the model (2005–2006 / 2007–2008 / 2009–2011). All tests of significance were two-sided, and *p* values < 0.05 were considered to denote significant differences. All analyses were done with SAS software version 9.3.

## Results

### Characteristics of study subjects at initiation of the new drug

Between 2005 and 2011, out of 77,488 individuals receiving combined antiretroviral therapy 54,847 individuals had at least one VL > 50 copies/mL. Among the 6049 individuals who were heavily pretreated, 5148 individuals started a new drug (as defined above). Among them, 4529 individuals had at least 6 months of follow-up after starting the new drug. Finally, 3884 individuals with available CD4 cell value obtained within 6 months before inclusion were enrolled in the study. Their median age was 44.8 years [interquartile range (IQR): 40.6–50.5] and they were at an advanced stage of HIV disease with a median CD4 cell count of 270/mm^3^ [IQR: 138–435] and a median VL of 3.90 log_10_ copies/mL [IQR: 2.81–4.80]. They had already been exposed to a median of 10 ARV [IQR: 8–13] and 42.8% had experienced an AIDS-defining event. Their median duration of exposure to ARVs was 11.4 years [IQR: 9.3–13.7]. There were 3046 individuals never exposed to ETR + PI, 2 individuals exposed to only ETR + PI and 836 individuals initially unexposed and then exposed to ETR + PI. Finally, 838 individuals (21.6%) were exposed to ETR + PI with darunavir (DRV) as the combined boosted PI in 82.5% of cases, boosted lopinavir in 7.1%, boosted atazanavir in 4.1%, boosted tipranavir in 3.9%, saquinavir in 1.3% and fosamprenavir in 1.1% of cases. ETR + PI was prescribed with RAL in 67.2% of cases, with T20 in 13.2%, with maraviroc in 4.6%, with NRTI in 58.7% of cases. As shown in Table 1, ETR + PI exposed patients tended to have more advanced HIV disease (in terms of AIDS status and the CD4 cell count), and to have been exposed to more ARV.

**Table 1:** Characteristics of individuals exposed and not exposed to ETR + PI contributing to follow-up, measured in number of persons-years

All patients (person-years)	All patients	No ETR + PI	ETR + PI	*p*
	13,985.68	11,666.71	2318.96	
Age at baseline				
18–29 years	333.96 (2.4%)	286.02 (2.5%)	47.94 (2.1%)	< 0.0001
30–39 years	3050.51 (21.8%)	2699.57 (23.1%)	350.94 (15.1%)	
40–59 years	9754.3 (69.7%)	7973.02 (68.3%)	1781.28 (76.8%)	
>=60 years	846.91 (6.1%)	708.10 (6.1%)	138.81 (6.0%)	
Gender and transmission group				
Men who have sex with men	5462.59 (39.1%)	4392.05 (37.6%)	1070.41 (46.2%)	< 0.0001
Injecting drug users	2393.23 (17.1%)	2087.84 (17.9%)	305.39 (13.2%)	
Other men	2788.01 (19.9%)	2258.16 (19.4%)	529.94 (22.8%)	
Other women	3341.84 (23.9%)	2928.67 (25.1%)	413.17 (17.8%)	
Geographic origin				
Sub-Saharan Africa	1218.56 (8.7%)	970.62 (8.3%)	247.94 (10.7%)	0.0002
Other	12,767.12 (91.3%)	10,696.10 (91.7%)	2071.02 (89.3%)	
HCV co-infection status				
No	11,650.70 (83.3%)	9643.24 (82.7%)	2007.47 (86.6%)	< 0.0001
Yes	2334.96 (16.7%)	2023.47 (17.3%)	311.49 (13.4%)	
Centre - Total follow-up of heavily treated individuals				
< 140 P-Y	2575.17 (18.4%)	2118.09 (18.2%)	457.02 (19.7%)	< 0.0001
140–240 P-Y	3254.79 (23.3%)	2717.26 (23.3%)	537.53 (23.2%)	
240–400 P-Y	3895.42 (27.9%)	3354.69 (28.8%)	540.73 (23.3%)	
≥ 400 P-Y	4260.31 (30.5%)	3476.68 (29.8%)	783.63 (33.8%)	
Calendar period				
2005–2006	8760.93 (62.6%)	8517.74 (73.0%)	243.19 (10.5%)	< 0.0001
2007–2008	3779.28 (27.0%)	2280.19 (19.5%)	1499.09 (64.6%)	
2009–2011	1445.46 (10.3%)	868.78 (7.4%)	576.68 (24.9%)	
Nadir CD4 count (cells/mm^3^) at baseline				
< 50	5090.31 (36.4%)	3973.69 (34.1%)	1116.62 (48.2%)	< 0.0001
50–100	2395.85 (17.1%)	1989.13 (17.0%)	406.72 (17.5%)	
100–200	3564.30 (25.5%)	3075.84 (26.4%)	488.46 (21.1%)	
≥ 200	2935.22 (21.0%)	2628.05 (22.5%)	307.16 (13.2%)	
CD4 count (cells/mm^3^) at baseline				
< 200	4879.74 (34.9%)	3871.50 (33.2%)	1008.24 (43.5%)	< 0.0001
200–350	4083.24 (29.2%)	3521.03 (30.2%)	562.21 (24.2%)	
350–500	2537.01 (18.1%)	2173.42 (18.6%)	363.59 (15.7%)	
≥ 500	2485.68 (17.8%)	2100.76(18.0%)	384.92 (16.6%)	
VL (copies/mL) at baseline				
50–500	2824.38 (20.2%)	2301.04 (19.7%)	523.34 (22.6%)	< 0.0001
500–5000	3255.58 (23.3%)	2763.23 (23.7%)	492.35 (21.2%)	
5000–30,000	2930.25 (21.0%)	2408.75 (20.6%)	521.50 (22.5%)	
30,000–100,000	2501.51 (17.9%)	2059.86 (17.7%)	441.65 (19.0%)	
> 100,000	2473.97 (17.7%)	2133.85 (18.3%)	340.12 (14.7%)	
Previous AIDS defining events at baseline				
No	8274.24 (59.2%)	7116.34 (61.0%)	1157.90 (49.9%)	< 0.0001
Yes	5711.44 (40.8%)	4550.31 (39.0%)	1161.06 (50.1%)	
Prophylaxis (Cotrimoxazole, Dapsone, Pentamidine)				
Not eligible (CD4 > 200)	8093.10 (57.9%)	6969.47 (59.7%)	1123.63 (48.5%)	< 0.0001
No	1821.36 (13.0%)	1378.35 (11.8%)	443.01 (19.1%)	
Yes	4071.22 (29.1%)	3318.89 (28.5%)	752.33 (32.4%)	
Number of prior ARVs				
5–8	3749.64 (26.8%)	3609.96 (30.9%)	139.69 (6.0%)	< 0.0001
9–10	3816.28 (27.3%)	3520.84 (30.2%)	295.44 (12.7%)	
11–13	4084.10 (29.2%)	3281.11 (28.1%)	802.99 (34.6%)	
> 13	2335.66 (16.7%)	1254.81 (10.8%)	1080.85 (46.6%)	

### Hospitalization rate and relative risks

**Fig. 1:**
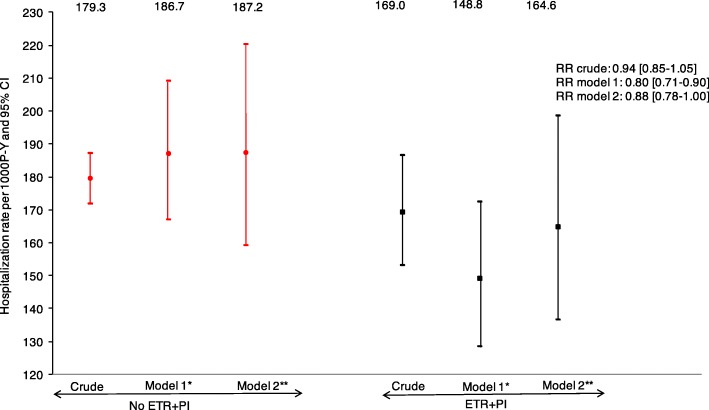
Crude and adjusted hospitalization rates and 95% confidence intervals from Poisson regression models according to ETR + PI exposure. *Adjusted on gender and transmission group, geographic origin, age, HCV co-infection status, the nadir CD4 cell count, the CD4 cell count, viral load, AIDS status at baseline, pneumocystis jiroveci prophylaxis, number of previous ARV and centre total follow-up of heavily treated individuals. **Additionnaly adjusted on NRTI and raltegravir use

During 13,986 persons-years of follow-up, there were 2484 hospitalizations in 956 individuals. As shown in Fig. 1, the crude hospitalization rates were 169.0 per 1000 P-Y for ETR + PI exposed individuals, versus 179.3 per 1000 P-Y for non-exposed individuals (RR = 0.94, 95% confidence interval: [0.85–1.05]). The adjusted hospitalization rates were 148.8 per 1000 P-Y for ETR + PI exposed individuals and 186.7 per 1000 P-Y for non-exposed individuals, corresponding to a 20% reduction (RR_adj_ = 0.80 [0.71–0.90]). After further adjustment for RAL and NRTI exposure, the estimated hospitalization rates were respectively 164.6 and 187.2 per 1000 P-Y, corresponding to a 12% reduction (RR_adj_ = 0.88 [0.78–1.00). In the sensitivity analysis, further adjusted on the calendar period, results were similar with an 18% reduction in the estimated hospitalization rates in ETR + PI exposed patients (RR_adj_ = 0.82 [0.72–0.94]).

### Hospitalization from a AIDS defining cause rate and relative risks

**Fig. 2:**
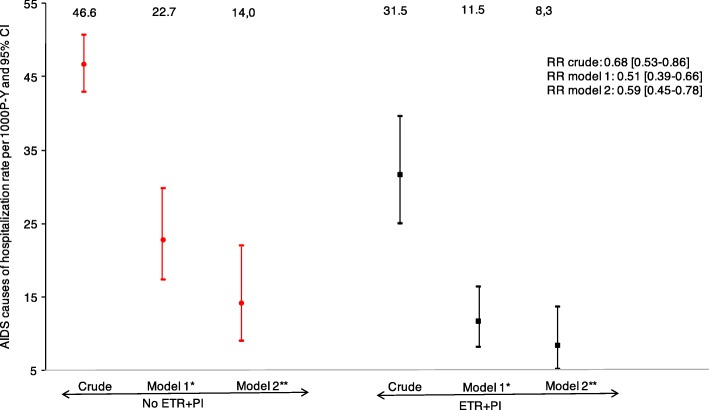
Crude and adjusted hospitalization from a AIDS defining cause rates and 95% confidence intervals from Poisson regression models according to ETR + PI exposure. *Adjusted on gender and transmission group, geographic origin, age, HCV co-infection status, the nadir CD4 cell count, the CD4 cell count, viral load, AIDS status at baseline, pneumocystis jiroveci prophylaxis, number of previous ARV and centre total follow-up of heavily treated individuals. **Additionnaly adjusted on NRTI and raltegravir use

During 13,986 persons-years of follow-up, there were 617 hospitalizations from an AIDS defining cause in 301 individuals. As shown in Fig. 2, the crude AIDS hospitalization rates were 31.5 per 1000 P-Y for ETR + PI exposed individuals, versus 46.6 per 1000 P-Y for non-exposed individuals (RR = 0.68, 95% confidence interval: [0.53–0.86]). The adjusted AIDS hospitalization rates were 11.5 per 1000 P-Y for ETR + PI exposed individuals and 22.7 per 1000 P-Y for non-exposed individuals, corresponding to a 49% reduction (RR_adjusted_ = 0.51 [0.39–0.66]). After further adjustment for RAL and NRTI exposure, the estimated AIDS cause of hospitalization rates were respectively 8.3 and 14.0 per 1000 P-Y, corresponding to a 41% reduction (RR_adjusted_ = 0.59 [0.45–0.78). In the sensitivity analysis, further adjusted on the calendar period, results were similar with a 46% reduction in the estimated AIDS cause of hospitalization rates in ETR + PI exposed patients*.* (RR_adjusted_ = 0.54 [0.41–0.73]).

### Hospitalization from a non-AIDS defining cause rate and relative risks

**Fig. 3:**
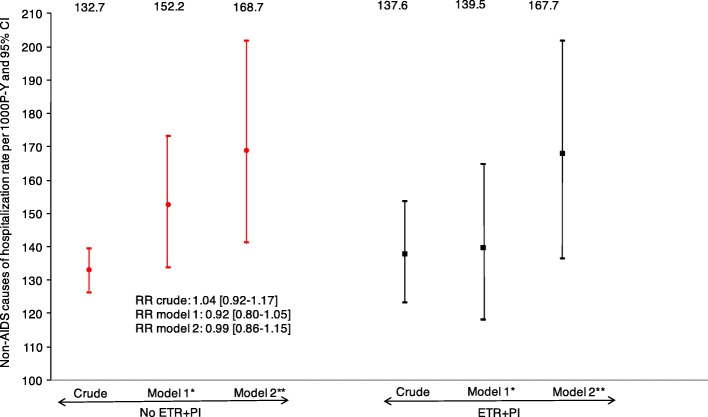
Crude and adjusted hospitalization from a non-AIDS defining cause rates and 95% confidence intervals from Poisson regression models according to ETR + PI exposure. *Adjusted on gender and transmission group, geographic origin, age, HCV co-infection status, the nadir CD4 cell count, the CD4 cell count, viral load, AIDS status at baseline, pneumocystis jiroveci prophylaxis, number of previous ARV and centre total follow-up of heavily treated individuals. **Additionnaly adjusted on NRTI and raltegravir use

During 13,986 persons-years of follow-up, there were 1867 hospitalizations from a non-AIDS defining cause in 828 individuals. As shown in Fig. 3, the crude non-AIDS hospitalization rates were 137.6 per 1000 P-Y for ETR + PI exposed individuals, versus 132.7 per 1000 P-Y for non-exposed individuals (RR = 1.04, 95% confidence interval: [0.92–1.17]). The adjusted non-AIDS hospitalization rates were 139.5 per 1000 P-Y for ETR + PI exposed individuals and 152.2 per 1000 P-Y for non-exposed individuals (RR_adjusted_ = 0.92 [0.80–1.05]). After further adjustment for RAL and NRTI exposure, the estimated non-AIDS cause of hospitalization rates were respectively 167.7 and 168.7 per 1000 P-Y (RR_adjusted_ = 0.99 [0.86–1.15). Similarly, the sensitivity analysis including the calendar period, did not show any significant difference in the non-AIDS cause of hospitalization rates (RR_adjusted_ = 0.94 [0.81–1.09]) between the two groups.

## Discussion

This study showed that, in routine clinical practice, heavily treated HIV-1-infected individuals who were exposed to ETR + PI between 2005 and 2011 had a lower risk of hospitalization than individuals who were not exposed to ETR + PI. This lower risk of hospitalization among individuals who were exposed to ETR + PI was mainly due to the lower risk of AIDS hospitalization. This study found a similar risk of hospitalization for a non-AIDS defining cause in the 2 groups*.* As virologic efficacy is a strong predictor of reduced clinical progression, the clinical benefit of ETR found in this study could be explained by a high rate of virologic suppression (62% at month 6) and excellent tolerability of this regimen [6, 8]. In the Duet-1 and Duet-2 trials ETR proved effective (57% of individuals had viral load < 50 copies/mL) and well tolerated at week 96 among treatment-experienced individuals [9]. Another French study also showed that ETR had good virologic efficacy at month 12 in heavily ART-experienced individuals (72% of individuals had viral load < 50 copies/mL) [10]. The efficacy of ETR could be explain by higher barrier than first-generation NNRTIs, against the development of drug resistance. In the DUET studies, the presence of the most common NNRTI mutation K103 N did not affect virological response to ETR [9]. When we adjusted for the use of RAL, the impact of ETR + PI on the risk of hospitalization was smaller but still statistically significant. The ANRS 139 Trio trial showed that an antiretroviral regimen containing ETR, RAL and DRV was well tolerated by treatment-experienced individuals with multidrug-resistant HIV infection and that it was associated with virologic suppression in 88% of cases at week 96 [11]. This could explain why the impact of ETR + PI on the risk of hospitalization was smaller, after taking RAL into account. Duet-1 and Duet-2 trials ETR did not evidence a significant difference in the risk of AIDS or death over 96 weeks in univariable analysis and showed a 36% reduction in multivariable analysis [9].

To our knowledge, our study is the first having focused on the risk of hospitalizations in current clinical practice and to show a positive effect, mainly due to a decrease in risk of AIDS hospitalizations. The main strengths of this study were the large individual population and the use of multivariable Poisson regression models with adjustement for confounding factors in an observational setting. As in any observational study, some residual confounding factors might remain. However, individuals exposed to ETR + PI in this study tended to have more advanced HIV disease and to have been exposed to a larger number of antiretroviral drugs, meaning that the impact of ETR + PI on the risk of hospitalization rate might have been underestimated. We were unable to adjust the analysis for the genotypic susceptibility score (GSS), which was not recorded in the FHDH. However, a European cohort study suggested that the baseline GSS had little influence on the virologic response to ETR [12]. The same study also showed that the GSS was higher in individuals receiving ETR and DRV than in those receiving ETR plus another PI. As most individuals in our study received DRV, genotypic ETR resistance was probably not a major issue. Similarly, the adherence was not recorded in the FHDH, but given that ETR is given twice-daily, which may lead to slightly lower adherence than once-daily regimen [13], adherence is unlikely to explain the better outcome with ETR.

## Conclusions

Our study showed that availability of ETR was associated with less frequent hospitalization among heavily treated HIV-1-infected individuals, showing the public health benefit of this drug. This result reinforces the need for new potent ARV active on multiresistant viral strains for heavily treated HIV-1-infected individuals on failing regimen.

## Abbreviations

ANRS: Agence Nationale de Recherches sur le Sida et les hépatites virales
ART: AntiRetroviral therapy
ARV: Antiretroviral
cART: combination AntiRetroviral therapy
DRV: Darunavir
ETR: Etravirine
FHDH: French Hospital Database on HIV
GSS: Genotypic susceptibility score
IQR: Interquartile range
NNRTI: Non-nucleoside reverse transcriptase inhibitor
NRTI: Nucleoside reverse transcriptase inhibitor
PI: Protease inhibitor
P-Y: Person-years
RAL: Raltegravir
RR: Relative risk
VL: Viral load
